# Brain natriuretic peptide to predict successful liberation from mechanical ventilation in critically ill patients: the results need to be interpreted with more caution

**DOI:** 10.1186/s13054-020-03106-y

**Published:** 2020-07-09

**Authors:** Chenyu Fan

**Affiliations:** Department of Emergency, Tongji Hospital of Tongji University, 389#Xincun Road, Shanghai, 200065 China

Dear Editor,

We read the recent article by Deschamps et al. [1], who designed a meta-analysis to conclude that the relative change in brain natriuretic peptide (BNP) during a spontaneous breathing trial (SBT) has potential value as an incremental tool to predict successful liberation from mechanical ventilation (MV) in adults. It is an exciting conclusion because MV is wildly used for critically ill patients during COVID-19. Nevertheless, we believe that the results need to be drawn with more caution.

Firstly, in this manuscript, both BNP and NT-proBNP were referred to as BNP. Although both of them can be responsive to the left ventricular afterload, they have different biological half-lives, molecular sizes, metabolites, renal clearance status, and stability, and the results of BNP and NT-proBNP cannot be compared directly. Moreover, BNP has poor stability in vitro and degrades rapidly when stored at room temperature and 4 °C. The stability of NT-proBNP is much better than that of BNP (25 °C can be stable for 7 days) [2]. Therefore, NT-proBNP may be a better prediction indicator for hospitals without proper test measures and need to be analyzed separately.

Secondly, there was considerable heterogeneity among the research objects included in the meta-analysis. Most studies reported a mixed population and included patients with heart disease and renal failure, which can significantly affect BNP’s baseline values. Besides, age is also an essential factor affecting the level of BNP [3]. When diagnosing acute heart failure, different BNP diagnostic threshold values are adopted for patients of different ages [4]. Consequently, pre-SBT BNP and post-SBT BNP are not good factors to predict liberation from MV, and the relative change in BNP during a SBT (ΔBNP%) only partially reflects the physiological changes before and after liberation because of diverse clinical features in patients. We believe that maybe the results are statistically significant in the mixed medical/surgical ICU population. However, it is less meaningful for each individual.

Finally, there were two other questions to discuss. Figures 4 and 5 showed the upper limit of the 95% confidence interval of ROC greater than 1. Though, the value of ROC is usually a range from 0.5 to 1 [5]. In Fig. 6, the two subheadings should be exchanged, and the first four studies were about post-SBT BNP.

Therefore, we think that the authors’ work is fascinating, but the meta-analysis results need to be interpreted with more caution, which was limited by the quality of the included literature.

## Authors’ response

Jean Deschamps

Dear Editor,

We thank our colleague Dr. Fan for his comments on our study [1]. We appreciate the additional insights provided on the complex use of different assays and heterogeneous populations included in this meta-analysis.

Dr. Fan has astutely noted that the upper limit of the 95% confidence interval of AUROC was greater than 1 in Figs. 4 and 5. These secondary analyses represent pooled estimated AUROC and are limited by the data provided in each individual study. Without the raw data, pooling of the AUROC of each study could only be performed using the provided AUROC and its standard error or 95% confidence interval. As such, this can result in confidence intervals that exceed the possible bound given that the normal distribution used for calculation is unbounded [6]. We felt it was appropriate to include these values since the standard errors simply determine the weight of each study in the pooled analysis and the final pooled analysis does not exceed the bounds.

Dr. Fan is also right regarding the inaccurate headings of Fig. 6. These were indeed inverted. We will provide an erratum regarding this.

Cordially,

Jean Deschamps, MDCM FRCPC

## Data Availability

Not applicable.

## Abbreviations

MV: Mechanical ventilation
BNP: Brain natriuretic peptide
SBT: Spontaneous breathing trial
ΔBNP%: Relative variation of BNP during a SBT
